# Teachers’ stress and training in a school-based mindfulness program: Implementation results from a cluster randomized controlled trial^☆^

**DOI:** 10.1016/j.jsp.2024.101288

**Published:** 2024-06

**Authors:** Summer S. Braun, Mark T. Greenberg, Robert W. Roeser, Laura J. Taylor, Jesus Montero-Marin, Catherine Crane, J. Mark G. Williams, Anna Sonley, Liz Lord, Tamsin Ford, The MYRIAD Team, Willem Kuyken

**Affiliations:** a Department of Psychology and Center for Youth Development and Intervention, The University of Alabama, McMillan Building, P.O. Box 870348, Tuscaloosa, Alabama, 35487, USA; b Department of Human Development and Family Studies, The Pennsylvania State University, 119 Health and Human Development Building, University Park, Pennsylvania, 16802, USA; c Department of Psychiatry, Oxford University, Warneford Hospital, Oxford, OX3 7JX, United Kingdom; d Parc Sanitari Sant Joan de Déu, C/ del Dr. Antoni Pujadas, 42, 08830 Sant Boi de Llobregat, Barcelona, Spain; e Department of Psychiatry, University of Cambridge, Douglas House 18b, Trumpington Road, Cambridge, CB2 8AH, United Kingdom

**Keywords:** Teachers, Mindfulness-based intervention, School-based intervention, Implementation quality, Teacher well-being

## Abstract

School-based mindfulness trainings (SBMT) are a contemporary approach for intervening to promote students' social and emotional skills and well-being. Despite evidence from the larger field of evidence-based social and emotional learning programs demonstrating the importance of high-quality implementation, few studies have investigated factors impacting the implementation of SBMTs, particularly teacher-level influences. The present study addressed this issue by investigating whether teachers' stress, trust in their fellow teachers and principal, and expectations about the program at baseline predicted the quality of their implementation of a SBMT for students. In addition, we examined whether teachers' stress at baseline moderated the effect of training condition on implementation quality. Implementation quality was assessed via observations and teacher self-reports. Results from a sample of British secondary (middle-high) school educators (*N* = 81) indicated that teachers who felt more supported by their principals at baseline were later observed to implement the SBMT with greater quality, whereas teachers who had more positive expectations about the program felt more confident teaching the course in the future. Teachers' baseline stress moderated the effect of training condition on all measures of implementation quality; among teachers experiencing high stress at baseline, more intensive training led to higher quality implementation. Implications for practitioners and prevention researchers are discussed.

## 1 Introduction

Effective implementation of evidence-based intervention programs for students is critical for producing their targeted outcomes (Domitrovich et al., 2010; Durlak & DuPre, 2008; Kam et al., 2003; Tudor et al., 2022). Unfortunately, there is substantial variation in the implementation of such programs in schools (Hicks et al., 2014; Ringwalt et al., 2009; Sanetti & Collier-Meek, 2019; Sanetti & Kratochwill, 2009; Witt et al., 1997). To advance the effective delivery of interventions, it is essential to understand the factors influencing implementation. Mindfulness-based programs for students have been gaining popularity in recent decades, yet few studies have investigated factors impacting implementation of programs employing this intervention approach (Felver & Jennings, 2016; Roeser et al., 2023). The present study addressed teacher-level influences that are thought to affect program implementation by investigating how teachers' baseline stress, occupational health, and expectations about the program predicted the quality of their implementation of a school-based mindfulness training (SBMT) for students and whether stress moderated the effect of training condition on implementation quality.

### 1.1 The importance of implementation in evidence-based programs for students

Evidence-based programs are those that have undergone rigorous scientific testing and are found to have beneficial effects for the target population (What Works Clearinghouse, 2017). Evidence-based social and emotional learning programs for students often involve a series of sequential didactic lessons and activities to cultivate students' social and emotional skills. These programs are known to benefit children in many ways, resulting in (a) improved social and emotional skills, (b) more positive attitudes towards self and others, (c) increased positive social behavior, (d) enhanced mental health, (e) improved academic performance, and (f) increased prevention of conduct problems and emotional distress (Durlak et al., 2011). Indeed, these programs can have effects long after program participation and even in areas not directly targeted in intervention (e.g., graduation rates; Taylor et al., 2017). Although SEL programs vary in targeted outcomes, content, formats, and delivery of the intervention, research shows that such programs overall have positive effects for youth (Durlak et al., 2011). Despite their potential usefulness, effective implementation of evidence based SEL programs is critical to their success. A meta-analysis of 542 studies of such programs for youth concluded that implementation had profound effects on outcomes; programs implemented well resulted in effect sizes several times higher than those with poorer implementation (Durlak & DuPre, 2008). Yet, despite their potential convenience for improving public health, school-based interventions are often at risk for poor implementation (Domitrovich et al., 2008; Sanetti et al., 2014). The emergence of the field of implementation science has brought an explicit focus on understanding, and in turn, addressing, the barriers that jeopardize effective implementation of such programs (Durlak, 2015; Eccles & Mittman, 2006).

Mindfulness-based interventions are a relatively new approach for promoting students' social and emotional health and well-being. These programs aim to cultivate students' mindfulness skills, or the active, nonjudgmental awareness of the present moment (Kabat-Zinn, 2003) through breath practices and other mindfulness-based experiential activities such as body scans and loving kindness meditations, among others. Recent evidence has concluded that such approaches can improve mindfulness and self-regulation skills, and these skills in turn show some promise for reducing feelings of stress, anxiety, depression, and self-harm, and supporting healthy relationships (Britton et al., 2014; Dunning et al., 2019; Kander et al., 2024; Kang et al., 2018; Roeser et al., 2023; Zenner et al., 2014). Yet, much remains unknown especially regarding how implementation affects outcomes (Jennings, 2023; Kuyken et al., 2022a; Tudor et al., 2022). Indeed, assessments of the factors influencing implementation of SBMTs are relatively rare, and when included, indicate substantial heterogeneity in implementation (Emerson et al., 2020; Gould et al., 2016; Zenner et al., 2014). To date there is no research on what teacher-related factors may influence the quality of implementation of SBMT programs. A small body of research has examined the association between the implementation of SBMTs and outcomes suggesting that greater dosage, measured as either attendance at program sessions or time devoted to outside practice, is related to superior program outcomes, but this research is scarce (Gould et al., 2016; Klingbeil et al., 2017). Thus, given the increasing prevalence of SBMT programs (Roeser et al., 2023) and evidence from the larger field of youth interventions emphasizing the importance of implementation (Durlak & DuPre, 2008), the present study investigated several factors theoretically related to teachers' implementation of a SBMT for students.

### 1.2 Conceptualizing implementation

Implementation refers to content of the program and how it is delivered in a specific setting (Durlak & DuPre, 2008). Durlak and DuPre described eight dimensions of implementation in their meta-analysis:

1. *Fidelity* is the extent to which a program aligns with the originally intended curriculum, also referred to as adherence or compliance.
2. *Dosage* refers to the amount of the original program that was provided, often measured by the number of program sessions delivered.
3. *Quality* is how well, clearly, and correctly the program was delivered.
4. *Participant responsiveness* is the extent to which the program stimulates interest and garners the attention of the participants.
5. *Program differentiation* refers to the uniqueness of the program from other interventions.
6. *Monitoring of comparison conditions* is the documentation of the services received by those outside of the intervention group; with researchers primarily focused on the intervention condition, the control group often goes unmonitored, yet knowing the activities of both groups is important when drawing conclusions about the comparative effect of a program.
7. *Program reach* refers to the proportion of involvement of individuals in a population and the representativeness of program participants, which is particularly important when considering program scale-up.
8. *Adaptation* refers to the changes made to the program that result in differences between that implementation and the original intervention.

Most implementation research has focused on the two dimensions of fidelity and dosage. Little is known about the effects of these other dimensions on program outcomes, especially in the context of SBMT programs (Gould et al., 2016; Roeser et al., 2023). Thus, the present study focused on implementation quality, investigating aspects of the implementer and program training that may impact teachers' quality of implementation of a SBMT.

### 1.3 Factors theorized to impact implementation

With the importance of implementation for program outcomes established, research has shifted to focus on understanding the factors or conditions that foster or jeopardize implementation. As many programs for youth are delivered in school settings, Domitrovich et al. (2008) offered a multi-level conceptual framework outlining factors influencing the implementation of school-based interventions to guide this research. Informed by ecological systems models (e.g., Bronfenbrenner, 1994), the Domitrovich et al. conceptual model posited that the implementation of school-based programs is impacted by an array of influences specific to the context in which the program is being implemented. These influences are described in three main categories, including (a) individual-level factors relating to the program implementer (e.g., occupational health, perceptions of the program), (b) school-level factors (e.g., school culture, resources), and (c) macro-level factors (e.g., federal, state, and district policies). Because a majority of SBMTs appear to be implemented by classroom teachers (vs. an external provider such as a clinician or mindfulness trainer; Roeser, Galla, & Baelen, 2022), understanding the role that teachers play in impacting implementation is of particular importance for SBMTs. As such, the present study focused on how characteristics of the teachers implementing the intervention may impact the quality of implementation of the program, which is what Domitrovich et al. (2008) termed individual-level factors. We focused on teachers' feelings of stress, teacher and principal trust, and expectations about the program due to their theoretical and empirical relevance to teachers' capacity to implement intervention programing for their students.

#### 1.3.1 Stress

**Fig. 1:**
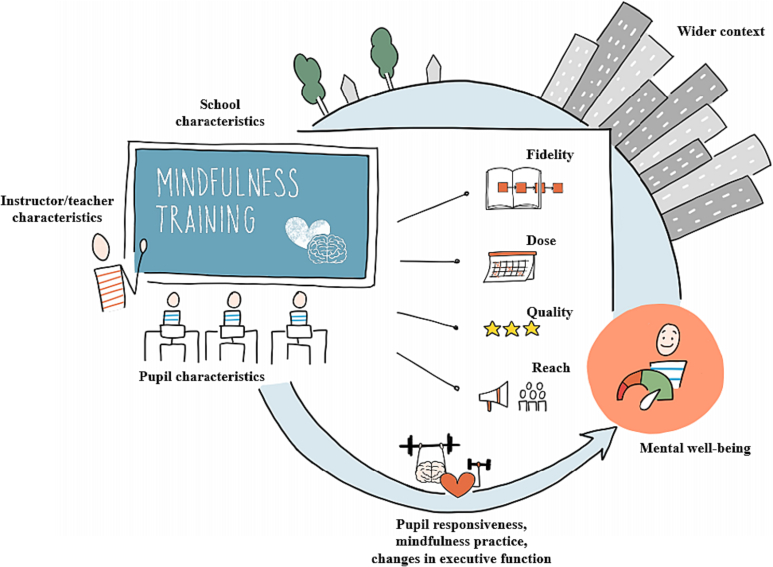
Conceptual Model of School-Based Mindfulness Trainings.

The Prosocial Classroom model (Jennings & Greenberg, 2009) and the conceptual model of SBMT (Fig. 1; Tudor et al., 2022) both posit that teachers' occupational health and well-being likely supports effective implementation, whereas feelings of stress jeopardize it. Theoretically, when teachers are under stress, their emotional resources, attention, and cognitive energy are devoted to coping, thereby leaving fewer resources for maintaining healthy relationships with students, supporting student learning, and effectively implementing programs (Boekaerts, 1993; Roeser et al., 2012; Roeser, Mashburn, et al., 2022). Research from the broader field of social and emotional learning (SEL) has supported these conceptual models; teachers' feelings of burnout, which emerge from chronic experiences of stress (Maslach et al., 2001), have been related to lower dosage implementation of several different school-based programs (Domitrovich et al., 2015; Ransford et al., 2009; Swift et al., 2017). In addition to diverting attention away from program implementation, stress is theorized to erode one's ability to fully embody mindfulness, which is thought to be critical to one's ability to teach mindfulness to others (Crane et al., 2013; Hedrick et al., 2017; Schussler et al., 2023). Thus, understanding the association between teachers' stress and program implementation is of particular importance in the context of SBMT where embodiment of a mindful attitude and behavior are paramount (Crane et al., 2013; Hedrick et al., 2017; Schussler et al., 2023).

#### 1.3.2 Teacher and principal trust

Conversely, positive social experiences at school have been found to support implementation. Specifically, teachers' perceptions of the general supportiveness of their principal and colleagues have been positively associated with teachers' dosage and quality of implementation of SEL programs (Cramer et al., 2021; Payne et al., 2006; Ransford et al., 2009). Evidence suggests that trusting, supportive relationships at school are also important in SBMT programs: Supportive relationships with school leadership and colleagues have been explicitly raised as factor that supported teachers' implementation of SBMTs in qualitative interviews (Joyce et al., 2010; Wilde et al., 2019), yet we know of no studies that have empirically investigated the role of teacher and principal trust in teachers' implementation of a SBMT.

#### 1.3.3 Program expectations

Teachers' perceptions and beliefs about the program and its effectiveness itself appear important for implementation. In the context of SEL programs, teachers with more negative views of the program at the outset (e.g., quality of the training) or beliefs contrary to the program's approach have lower implementation dosage and quality than teachers with more positive perceptions (Cook et al., 2015; Ransford et al., 2009). There is initial evidence that this element is also important in SBMTs (Wilde et al., 2019).

#### 1.3.4 A confluence of influence

The present study aimed to extend the research on implementation of programs for students by focusing on teachers' implementation of a SBMT and examining the influence of teachers' stress, teacher and principal trust, and expectations of the program at baseline in predicting the quality of their implementation of the SBMT in the classroom. Teachers' experiences of stress are thought to be a barrier to high quality implementation, whereas supportive relationships with other teachers and their principal and positive perceptions of the program itself are thought to facilitate teachers' high-quality implementation of SBMTs. Yet, the study of implementation is more complex than identifying simple barriers and facilitators of a program (Lendrum & Humphrey, 2012). In their Interactive Systems Framework for Dissemination and Implementation, Durlak and DuPre (2008) posited that provider characteristics may interact with features of the program (e.g., program training) to influence implementation. Although most implementation research has focused on barriers and facilitators to implementation in a unidimensional way (Durlak & DuPre, 2008), the few studies that have assessed multiple factors did so in the context of predicting outcomes of SEL programs rather than implementation (Durlak & DuPre, 2008; Kam et al., 2003; Reyes et al., 2012). For example, Reyes et al. (2012) found that outcomes of a SEL program were affected by a combination of the intensity of teacher training and the quality with which they implemented the program. We are not aware of any studies that have embraced this nuanced approach in predicting teachers' implementation of SBMTs. In the context of the SBMT program of focus in the present study, recent research has demonstrated that teachers who participated in more intensive training experiences do not differ significantly in their implementation of the program (Crane, Ganguli, et al., 2020). However, null main effects may be masking a more complex association between training and teachers' implementation of SBMT. Because highly stressed teachers are theorized to be at risk for poor implementation (Jennings & Greenberg, 2009), we hypothesized that the implementation by stressed teachers would be better when they receive more intensive training in the SBMT as more intensive training is indicative of additional support that may be especially needed in this group. The association between teachers' stress and training condition in predicting program implementation is particularly relevant considering the training conditions support teachers' own personal mindfulness practice and delivery of the curriculum for students to varying degrees; a detailed description of the unique training procedures in the present study that informed this hypothesis are elaborated on in the subsequent section. Thus, the present study advances the field of implementation of SBMT by considering whether teachers' stress moderates the effect of program training on implementation quality whereby assessing *for whom* specific training regimens work best.

### 1.4 The MYRIAD project

The MY Resilience in Adolescence (MYRIAD) project involves a series of studies focused on the impact of a SBMT for secondary school students and their teachers. Existing findings from this project are reported in Crane, Ganguli, et al. (2020), Montero-Marin, Taylor, et al. (2021), Kuyken et al. (2022a, 2022b), and Montero-Marin et al. (2022). The present study reports findings from a study of scalability/implementation, which investigated several pathways to prepare teachers to deliver the SBMT for adolescents, the .b program (Mindfulness in Schools Project, 2021). All teachers first received personal mindfulness training (Phase 1) followed by training on the delivery of the .b curriculum for students in their classrooms (Phase 2). In each of the two phases there were two types of training experiences. Thus, after randomization was complete, teachers participated in one of four different training experiences (see Intervention Procedure for more details about the randomization procedure).

#### 1.4.1 Phase 1: Teacher personal mindfulness training

Training teachers in mindfulness was important because research from the larger field of interventions for youth demonstrates that familiarity with program content is related to teachers' implementation of the program (LaChausse et al., 2014). In addition, the personal mindfulness component for teachers was particularly important in the context of a SBMT—perhaps more so than other types of interventions for youth—because of the theorized connection between teachers' own capacity to fully embody mindfulness and their ability to teach mindfulness to others. It is theorized that teachers (and other mindfulness instructors) cannot successfully guide others in learning mindfulness without personal experience and commitment to mindfulness practice (Crane et al., 2013; Hedrick et al., 2017; Schussler et al., 2023). Especially because teaching mindfulness is not something teachers are usually exposed to in their education, the personal mindfulness training was an important first phase of the program.

Regarding the teacher's personal mindfulness training, teachers were randomized to either a self-taught or instructor-led personal mindfulness training. Both the self-taught and instructor-led trainings were based on Penman and Williams' (2011) book *Mindfulness: A Practical Guide to Finding Peace in a Frantic World*. The self-taught group was prompted to read the chapters weekly in keeping with the pacing of the in-person training. Teachers in the in-person training also read the book and participated in eight, 90-min group sessions led by an experienced mindfulness instructor. The dual aims of the personal mindfulness trainings were to (a) develop teachers' mindfulness skills and knowledge and (b) improve their occupational health and well-being (see Roeser et al., 2012). Results of this research are presented in Montero-Marin, Taylor, et al. (2021). In sum, although teachers in the Self-Taught condition demonstrated significant improvements over time in self-compassion and well-being, teachers in the Instructor-Led condition showed significantly greater improvements at post-test than teachers in the Self-Taught condition on measures of mindfulness, self-compassion, well-being, stress, anxiety, and depression.

#### 1.4.2 Phase 2: Curriculum training

After teachers' personal mindfulness training, teachers were trained to implement the .b (pronounced [dot-be], standing for “stop and be”) curriculum in their classrooms. The .b curriculum is a fully manualized, sequenced, 10-lesson (typically 30–50 min each), school-based mindfulness program for 11–18 year old students delivered by the classroom teacher over one school term (see www.mindfulnessinschools.org/teach-dot-b/dot-b-curriculum/ for more information). The program aims to teach mindfulness skills that support young people's resilience, using a combination of psychoeducation, class discussion, and mindfulness practices. It was adapted from mindfulness-based cognitive therapy to make it acceptable to young people across the full spectrum of functioning from mental health problems to flourishing. The intention is to introduce young people to a range of skills (e.g., attentional control, self-regulation of thoughts, feelings, and behaviors) that they are encouraged to use in their everyday lives.

Based on condition assignment, teachers' training in the .b curriculum occurred in a 1-day or a 4-day training format. The goal of these trainings was to support teachers in implementing the mindfulness curriculum for students in a high-fidelity, high-quality manner in their classrooms. The 4-day training is the standard teacher training route that usually accompanies the .b training. However, there are challenges with scheduling 4 days to train teachers and finding teachers to cover their lessons and work for this period. Thus, a succinct, 1-day training was created for the present study by the original program developers to explore whether the training could be taught effectively in a shorter time to ease the burden on schools. Both the 4-day and 1-day courses involved the modeling of program lessons (in vivo and through viewing videos of experienced teachers), experience leading mindfulness practice and enquiry, and periods of personal, teacher-focused mindfulness practice. Additionally, those in the 4-day training spent time in groups with the others from their school and participated in sessions about the implementation of the SBMT in their school. Results regarding the effect of training condition on teachers' mindfulness skills and implementation quality are published in Crane, Ganguli, et al. (2020), with the main finding that teachers in the Instructor-Led, 4-Day condition reported greater mindfulness skills but no significant differences in observed competency teaching the .b curriculum than teachers in the Self-Taught, 1-Day condition. Aside from the main effect of training condition, what remains unknown is whether teachers' baseline characteristics impact observed and self-reported implementation and whether teachers' stress may interact with training to influence implementation.

### 1.5 Present study

The present study extends the existing knowledge about the effects of the MYRIAD project by applying an implementation science perspective to investigate factors that may impact the quality of teachers' implementation of the SBMT for students. This study addressed two primary lines of inquiry. First, we examined whether teachers' baseline levels of stress, teacher and principal trust, and expectations of the program impacted the quality of their implementation of the SBMT as measured by observational and self-report measures above and beyond the impact of teachers' training experiences (i.e., training condition; Self-Taught or Instructor-Led personal mindfulness training, coupled with the 1-Day or 4-Day curriculum training). Second, we explored whether teachers' baseline levels of stress moderated the impact of training condition on their quality of implementation of the SBMT.

## 2 Method

### 2.1 Study design and recruitment

This study within the MYRIAD project was a randomized-controlled trial (RCT) of the teacher training aspect of the SBMT, the .b mindfulness program (Montero-Marin, Taylor, et al., 2021). The study was registered prior to participant consent and randomization (11/24/2015; ISRCTN18013311) and was reviewed and approved by the University of Oxford Medical Sciences Ethics Committee (03/20/2015, ref.: MS-IDREC-C1–2015- 048); the study was monitored by a Data Monitoring and Ethics Committee (DMEC; see Crane, Ganguli, et al., 2020, for additional information).

All secondary schools and Local Education Authorities in England (state, selective, and private; > 3000) were contacted via email (email addresses were identified through an existing freedom of information request) about participation in the study. Potential participating schools were also recruited in parallel via word of mouth, education events, and information placed on specialist education forums. All headteachers and teachers who were contacted were invited to express interest to the researchers via email. If schools met the inclusion and exclusion criteria for the study, they were invited to participate.

Schools were eligible if they were not receiving external support or interventions for a poor quality rating, had a permanent headteacher in place (not interim), had not delivered a mindfulness program in the last calendar year, were accessible to a mindfulness instructor (not geographically remote), would allow all participating teachers to attend personal mindfulness training and syllabus training for delivering the course to pupils, were able to schedule the pupil mindfulness curriculum within the school day to at least one class of pupils per teacher, and were able to commit at least three teachers to deliver mindfulness classes. Interested teachers within eligible schools were also screened for eligibility. Teachers were eligible to participate if they held qualified teacher status or had 5 years of teaching experience, could attend the personal mindfulness training (an 8-week course, delivered predominantly outside of school hours in their own school), could attend the syllabus training (up to 4 days) to deliver the mindfulness program to pupils, were not planning on leaving the teaching profession in the next 12–18 months, were a permanent member of staff, had not completed a personal mindfulness course in the last calendar year, and had not previously been trained to deliver a mindfulness course. After headteachers had consented for their school to participate, an email was sent to all eligible staff soliciting their participation. The final list of participating teachers was approved by the research lead and the headteacher. A teacher was considered a participant only after they had completed the baseline measures and a school was considered as participating after the headteacher consented and three consenting teachers had completed the baseline measures.

Responses were initially received from 185 schools. Following screening, there were 75 schools and 254 teachers who were eligible and interested. After exclusion of schools with fewer than three participating members of staff and schools located in a geographical region too logistically difficult to be reached by a mindfulness instructor, the final sample for the trial consisted of 206 teachers in four conditions (see Intervention Procedure below), across 43 schools (see Crane, Ganguli, et al., 2020, for the full participant flow). Participating teachers and schools were largely representative of English secondary schools with respect to gender, race, years teaching, and the population served on key measures such as deprivation, operationalized as the percentage of children eligible for free school meals, and the type of school (i.e., selective/non- selective, urban/rural, large/small, mixed/single gender, and state maintained/independent). For additional teacher and school characteristics, please see Crane, Ganguli, et al. (2020), Montero-Marin, Taylor, et al. (2021), and Kuyken et al. (2022a).

### 2.2 Intervention procedure

Participating teachers completed baseline surveys of their demographics, stress, and teacher and principal trust. The Peninsula Clinical Trials Unit then randomized schools to one of four conditions (see below), with two strata (schools with one to four recruited teachers and schools with five to nine recruited teachers). The intervention occurred in two phases consisting of personal mindfulness training (Phase 1) and training in how to deliver the SBMT curriculum for students (Phase 2). Regarding the first phase, schools were randomized for their teachers to receive support for their personal mindful awareness to either the self-taught or instructor-led personal mindfulness training groups, with both trainings based on Penman and Williams' (2011) book *Mindfulness: A practical guide to finding peace in a frantic world*. Teachers in the self-taught condition were asked to read the introductory chapters before the course started and then follow the eight course chapters and exercises weekly for 8 weeks. The instructor-led groups followed an 8-week face-to-face mindfulness course based on the book in groups of three to nine participants. Instructor-led sessions were delivered in each school (usually outside of school hours), lasting 90 min each and were led by trained, experienced mindfulness instructors. Teachers in both conditions were given similar start times to ensure that the two conditions were equally spread across the school term. In both conditions there were a range of home practice exercises and a daily 20-min mindfulness practice. Teachers in both conditions were also given access to a CD/MP3 of guided practices and access to an app that accompanies the course (see Montero-Marin, Taylor, et al., 2021, for more details about the course content). Two weeks into the personal training, teachers reported on their expectations of the personal mindfulness training. In Phase 2 of the study, teachers received either a 1-day or a 4-day training, based on randomization condition, in the Mindfulness in Schools Project .b classroom mindfulness curriculum. Both the 1-day and 4-day trainings took place in July 2016 at three locations in England. These courses were only attended by participating teachers who were trained alongside colleagues from their school (see Crane, Ganguli, et al., 2020, for more details about the training).

In sum, the randomization procedure resulted in a total of four intervention/training conditions, displayed in Table 1. Teachers in Condition 1 received the self-taught personal mindfulness training and the 1-day curriculum training. Teachers in Condition 2 received the instructor-led personal mindfulness training and the 1-day curriculum training. Teachers in Condition 3 received the self-taught personal mindfulness training and the 4-day curriculum training. Teachers in Condition 4 received the instructor-led personal mindfulness training and the 4-day curriculum training. After the personal mindfulness training and curriculum training were complete, teachers completed a post-intervention survey and submitted video recordings of their lessons. For a detailed description of the entire MYRIAD study, including the study protocol, randomization procedure, and CONSORT Diagram, please see Kuyken et al. (2017), Montero-Marin, Nuthall, et al. (2021), and Montero-Marin, Taylor, et al. (2021).

**Table 1:** Intervention/Training Conditions*.*

		**Personal Mindfulness Training**		
		Self-Taught		Instructor-Led
**Curriculum Training**	1-Day	Condition 1 (Self Taught, 1-Day)		Condition 2 (Instructor-Led, 1-Day)
	4-Day	Condition 3 (Self Taught, 4-Day)		Condition 4 (Instructor-Led, 4-Day)

### 2.3 Participants

The analytic sample included 81 educators who participated in the study. Because of our interest in post-intervention assessments of implementation quality, only teachers who completed surveys after the curriculum was implemented (i.e., at post-intervention) were included in these analyses (40% of total participants). This decision was made because differences between groups suggested systematic attrition, indicating that accounting for missing data in analytic modeling was not appropriate. Teachers who completed both surveys differed from teachers who were missing post-intervention surveys in several specific ways: Teachers with complete data had significantly higher baseline principal trust (*M*_Complete_ = 3.24, *M*_Missing_ = 3.00, t(204) = 2.63, *p* = .01) and somewhat higher teacher trust (*M*_Complete_ = 3.20, *M*_Missing_ = 3.07, t(193) = 1.85, *p* = .07) and expectations of the program (*M*_Complete_ = 7.27, *M*_Missing_ = 6.86, t(125) = 1.90, *p* = .06).

This resulted in an analytic sample of 81 secondary school educators (79% female; 14% racial minority; *M*_Experience_ = 13.31 years, *SD* = 8.61) from 31 schools, with one to eight teachers per school, with each school having an average of two to three teachers (*M*_Enrollment_ = 1014 students, *SD* = 361; *M*_*FreeSchoolMeals*_ = 27.26%, *SD* = 16.91%).

### 2.4 Measures

#### 2.4.1 Implementation quality outcomes

Teachers' quality of implementation of the .b curriculum was assessed at post-intervention in the following three ways: (a) observed teaching competence, (b) self-reported teaching confidence, and (c) self-reported confidence teaching the course in the future.

**Observed Teaching Competence.** Teachers' competence while teaching the .b curriculum was assessed via third-person observations of teachers using the Mindfulness-Based Interventions: Teaching Assessment Criteria (MBI:TAC; Crane et al., 2013; Crane & Kuyken, 2019) with wording adapted to be relevant to school teachers delivering mindfulness training to students (MBI:TAC TEACH). The MBI:TAC TEACH assesses teachers' quality of implementation of the .b curriculum in a series of six domains: (a) Domain 1: Coverage, pacing, and organization; (b) Domain 2: Relational skills; (c) Domain 3: Embodiment of mindfulness; (d) Domain 4: Guiding mindfulness practices; (e) Domain 5: Conveying course themes; and (f) Domain 6: Holding the group learning environment. Each domain is scored, after which a composite score is assigned which reflects teachers' overall competence across the domains on a scale of 1–6 (1 = *Incompetent*, 6 = *Advanced*). Because of our interest in teachers' general quality of implementation rather than domain-specific implementation, the composite score was analyzed in the present study. Raters were (a) independent of the training team (i.e., did not provide any support or supervision to teachers during the personal mindfulness nor curriculum training phases), (b) experienced mindfulness teachers, (c) experienced in the delivery of the .b program, and (d) trained to use the MBI-TAC; these individuals received additional training in the administration of the MBI-TAC TEACH addendum in a 2-day workshop. After training, the reliability of raters was assessed. Raters reviewed videos of three teachers and rated the competency of each. Their ratings were compared to benchmark values established by expert raters and an average disagreement score was calculated for each rater. All raters had an average discrepancy of less than one point on the MBI-TAC TEACH from the benchmarks, indicating a high degree of consistency between independent raters and the benchmark. During subsequent independent video coding, all raters participated in periodic video conferences to review processes and raise any issues arising during coding. After being trained and deemed reliable, raters watched each teachers' video twice, completing the MBI:TAC TEACH after the first observation and reassessing the score after the second observation, as necessary. With research demonstrating that the measure can be used to assess teachers' competence teaching mindfulness and that training results in high levels of interrater reliability, practitioners' and researchers' use of the MBI:TAC has increased in recent years (Crane, Hecht, et al., 2020). For more information about the MBI:TAC and MBI:TAC TEACH, please see Crane, Hecht, et al. (2020) and the online supplementary materials from Crane, Ganguli, et al. (2020).

**Self-Reported Teaching Competence.** Teaching the course was assessed using one item specifically designed for this study, “How competently do you feel you delivered the course?”. The item was rated on a 1–9 scale (1 = *Not at all competently*, 9 = *Very competently*). This brief measure was used for its practicality in this applied setting to minimize participant burden.

**Self-Reported Confidence Teaching the Course in the Future.** Teachers' confidence teaching the course in the future was assessed using one item specifically designed for this study, “How confident would you feel teaching the course again?” The item was rated on a 1–9 scale (1 = *Not at all confident*, 9 = *Very confident*). This brief measure was used for its practicality in this applied setting to minimize participant burden.

#### 2.4.2 Demographics

Teachers self-reported on their gender identity (0 = *Male*; 1 = *Female*) and randomization condition was noted.

#### 2.4.3 Baseline stress, teacher and principal trust, and program expectations

At baseline, teachers self-reported on their perceived stress, trust in their fellow teachers, and trust in their principal. Teachers' expectations of the program were assessed 2 weeks into the personal mindfulness training so that they had a basic understanding of what the program would entail. Alphas for the present sample are reported.

**Stress.** Teachers' stress was measured using 10 items from the Perceived Stress Scale (Cohen et al., 1983). Items (e.g., “In the last month, how often have you found that you could not cope with all the things that you had to do?”) were rated on a 1–5 scale (1 = *Never*, 5 = *Very Often*; α = 0.85). Items were reversed-scored when necessary such that higher values indicate greater stress, and items were averaged to create the scale score. Psychometric research using various samples has demonstrated that this measure has been reported to have adequate reliability (> 0.70) and validity as it is correlated with related experiences of depression and anxiety symptoms, among others (Cohen et al., 1983; Lee, 2012).

**Teacher Trust***.* Teachers' trust in their colleagues was assessed using six items from the Teacher Trust Scale (Bryk & Schneider, 2002). Items (e.g., “Teachers in this school trust each other”) were rated on a 1–4 scale (1 = *Strongly Disagree*, 4 = *Strongly Agree*; α = 0.87) and averaged to create the scale score. The measure is reported to have adequate reliability (α = 0.89) and validity (e.g., Consortium on Chicago School Research, 2003).

**Principal Trust.** Teachers' trust in their principal was assessed using nine items (e.g., “It's okay in this school to discuss feelings, worries, and frustrations with the headteacher”) from the Principal Trust Scale (Bryk & Schneider, 2002). Items were rated on a 1–4 scale (1 = *Strongly Disagree*, 4 = *Strongly Agree*; α = 0.93) and averaged to create the scale score. The measure is reported to have adequate reliability (α = 0.89) and validity (e.g., Sebastian & Allensworth, 2012).

**Program Expectations.** Teachers' expectations about the program were assessed using five items (e.g., “How much does what's being taught in this course make sense to you?”) adapted from a previous school-based mindfulness study (Bluth et al., 2016) drawn from the Credibility/Expectancy Questionnaire (Borkovec & Nau, 1972). Items were rated on a 1–9 scale (1 = *Not at all*, 9 = *Very*; α = 0.88) and averaged to create the scale score. The measure is reported to have adequate reliability (α > 0.80 for original measure; α = 0.87 for version adapted for mindfulness studies) and has been found to be predictive of later program outcomes (e.g., Bluth et al., 2016; Devilly & Borkovec, 2000).

### 2.5 Analytic strategy

A series of hierarchical linear regression models were run in R Studio to assess whether teachers' baseline stress, teacher and principal trust, and expectations about the program were associated with their quality of implementation of the .b program and whether teachers' baseline stress moderated the association between their assigned training condition and implementation quality. Cluster robust standard errors were estimated to account for nesting of teachers within schools, which is a method that results in standard errors that more accurately reflect the variability given the clustering than ordinary least squares regression (McNeish et al., 2017). Cluster robust standard errors were used rather than multilevel modeling due to the small number of schools/clusters (*N* = 31), the small number of teachers within each cluster (range = 1–8; i.e., in some cases there was no variation within schools because there was only one participating teacher at the school), and because the clustering of teachers in schools was necessary to accommodate rather than of substantive interest.

Three hierarchical models were run for each of the three implementation quality outcomes. Model 1 served as the basic model for comparison and included demographic variables only (i.e., teachers' gender and training condition). Training condition was dummy coded with Condition 1 (Self-Taught, 1-Day) as the reference group because this was the group with the minimal amount of training. To address the first question (i.e., whether teachers' initial levels of stress, teacher trust, principal trust, and program expectations were related to observations and self-reports of implementation quality, above and beyond the main effect of training condition), we ran Model 2, which included all variables in Model 1 and the main effects of teachers' reports of perceived stress, teacher trust, principal trust, and program expectations. These variables were grand mean centered such that the intercept in these models may be interpreted as the implementation quality for a male teacher in Condition 1 with an average level of stress, teacher trust, principal trust, and program expectations.

To address the second question (i.e., whether teachers' baseline levels of stress moderate the impact of the training on implementation quality of the .b program, above and beyond the main effect of training condition), in Model 3 we included all variables in Model 2 and the interaction of condition by stress. Because condition was dummy coded with Condition 1 as the reference group, this resulted in three additional parameters: Condition 2*Stress, Condition 3*Stress, and Condition 4*Stress, which allowed us to assess whether the effect of stress differed between teachers in Conditions 1 and 2, 1 and 3, and 1 and 4. At each step, a Wald test compared the model to the prior, more simplistic model, with a significant Wald test indicating whether the subsequent model was a significantly better fit for the data. Once the best fitting model was determined, the effects within that model were interpreted.

#### 2.5.1 Missing data

Teachers who did not complete post-intervention data collection were omitted from the analytic sample (see Participants for more information). Because these data were not missing at random, neither traditional nor contemporary approaches for accommodating missing data (e.g., listwise deletion, maximum likelihood estimate, multiple imputation) were appropriate (Croy & Novins, 2005). After this step, all remaining participants had complete data (no missing data on outcomes nor predictors) except for three participants who did not have data for observed teaching competence. As these cases constituted < 4% of the sample, listwise deletion was used in the models predicting observed teaching competence as dropping these cases was unlikely to result in biased estimates (Bennett, 2001).

## 3 Results

Descriptive statistics and correlations are provided in Table 2, Table 3, and Table 4. Model comparisons are presented in Table 5 and results for the best fitting models are presented in Table 6.

**Table 2:** Demographics of Analytic Sample*.*

	***N***	**%**
**Training Condition**		
Condition 1: Self-Taught, 1-Day	24	30%
Condition 2: Instructor-Led, 1-Day	11	14%
Condition 3: Self-Taught, 4-Day	17	21%
Condition 4: Instructor-Led, 4-Day	29	36%
**Gender**		
Male	17	21%
Female	64	79%
**Race**		
White British	70	86%
Other	11	14%

**Table 3:** Descriptive Statistics of Analytic Sample.

**Measure**	***N***	**% Missing**	***M***	***SD***	**Min**	**Max**	**Skew**
**Implementation Quality Outcomes**							
Observed Teaching Competence (MBI: TAC TEACH)	78	4%	2.63	0.87	1	4	0.08
Self-Reported Teaching Competence	81	0%	5.91	1.48	1	9	−1.01
Self-Reported Confidence Future Teaching	81	0%	7.26	1.34	3	9	−1.24
**Baseline Stress, Teacher and Principal Trust, and Program Expectations**							
Stress	81	0%	1.44	0.60	0.20	3.10	0.30
Teacher Trust	81	0%	3.18	0.52	1.83	4.00	−0.31
Principal Trust	81	0%	3.23	0.60	1.78	4.00	−0.63
Program Expectations	81	0%	7.27	1.22	2.00	9.00	−1.51

**Table 4:** Bivariate Correlations Among Teachers' Quality of Implementation of the .b Curriculum, Demographics, and Baseline Stress, Teacher and Principal Trust, and Program Expectations.

		**Implementation Quality Outcomes**			**Demographics**					**Baseline Stress, Teacher and Principal Trust, and Program Expectations**			
		1	2	3	4	5	6	7	8	9	10	11	12
	**Implementation Quality Outcomes**												
1	Observed Teaching Competence (MBI: TAC TEACH)	-											
2	Self-Reported Teaching Competence	0.18	-										
3	Self-Reported Confidence Future Teaching	0.17	0.60	-									
	**Demographics**												
4	Gender (1 = *Female*)	0.04	−0.05	−0.08	–								
5	Condition 1: Self-Taught, 1-Day	−0.11	−0.27	−0.13	0.00	–							
6	Condition 2: Instructor-Led, 1-Day	−0.12	0.17	−0.02	−0.15	−0.26	–						
7	Condition 3: Self-Taught, 4-Day	0.11	0.17	0.15	0.27	−0.33	−0.20	–					
8	Condition 4: Instructor-Led, 4-Day	0.11	−0.01	0.01	−0.12	−0.48	−0.30	−0.38	–				
	**Baseline Stress, Teacher and Principal Trust, and Program Expectations**												
9	Stress	−0.18	0.01	0.05	0.01	−0.22	−0.07	−0.01	0.26	–			
10	Teacher Trust	0.06	0.20	0.21	−0.03	0.00	0.00	0.02	−0.02	−0.34	–		
11	Principal Trust	0.29	0.04	0.12	−0.01	0.16	−0.22	0.04	−0.03	−0.09	0.46	–	
12	Program Expectations	0.20	0.06	0.28	−0.10	−0.16	−0.11	0.06	0.18	−0.09	0.14	0.16	–

*Note*. Correlations > |0.22| are significant at *p* < .05.

**Table 5:** Wald Tests Comparing Model Fit of Hierarchical Linear Models Predicting Teachers' Observed and Self-Reported Quality of Implementation of the .b Curriculum.

	**Observed Teaching Competence** **(MBI: TAC TEACH)**			**Self-Reported Teaching Competence**			**Self-Reported Confidence Future Teaching**	
	*X*^*2*^(df)	*p*-value		*X*^*2*^(df)	*p*-value		*X*^*2*^(df)	*p*-value
Model 1 vs. Model 2	*X*^2^(4) = 18.50	< 0.001		*X*^2^(4) = 6.56	0.16		*X*^2^(4) = 10.03	0.04
Model 2 vs. Model 3	*X*^2^(3) = 12.03	< 0.001		*X*^2^(3) = 11.96	0.01		*X*^2^(3) = 7.99	0.05
Model 1 vs. Model 3	*X*^2^(7) = 65.19	< 0.001		*X*^2^(7) = 24.54	< 0.001		*X*^2^(7) = 12.22	0.09

**Table 6:** Results of Best Fitting Models – Model 3 – in Predicting Teachers' Observed and Self-Reported Quality of Implementation of the .b Curriculum.

	**Observed Teaching Competence** **(MBI: TAC TEACH)**				**Self-Reported Teaching Competence**				**Self-Reported Confidence Future Teaching**		
	*B*		*SE*		*B*		*SE*		*B*		*SE*
Intercept	2.20	⁎	0.30		5.37	⁎	0.62		7.27	⁎	0.39
**Demographics**											
Gender (1 = *Female*)	0.11		0.26		−0.26		0.49		−0.27		0.35
Condition 2	0.18		0.29		1.37	⁎	0.62		0.10		0.42
Condition 3	0.50		0.34		1.30	⁎	0.61		0.59		0.40
Condition 4	0.43		0.28		0.54		0.62		−0.13		0.48
**Baseline Stress, Teacher and Principal Trust, and Program Expectations**											
Stress	−0.73	⁎	0.21		−0.65	⁎	0.29		−0.08		0.34
Teacher Trust	−0.44		0.24		0.50		0.30		0.43		0.40
Principal Trust	0.52	⁎	0.16		0.03		0.32		−0.01		0.30
Program Expectations	0.08		0.07		0.01		0.14		0.27	⁎	0.11
**Interactions**											
Condition 2⁎Stress	−0.30		0.21		0.71		0.44		−0.55		0.76
Condition 3⁎Stress	0.56		0.45		1.15	⁎	0.50		0.23		0.43
Condition 4⁎Stress	0.77	⁎	0.30		1.52	⁎	0.49		1.28	⁎	0.52
R^2^	0.27				0.21				0.23		

*Note*. Models used cluster robust standard errors to account for clustering of teachers within schools.

⁎ *p* < .05.

### 3.1 Observed teaching competence: MBI:TAC TEACH

Model 1 accounted for 4% of the variance in observers' ratings of teachers' quality of implementation of the .b curriculum, indicating that teachers' gender and training condition explained a small but nonsignificant amount of the variance in the MBI:TAC TEACH, *X*^2^(4) = 2.58, *p* = .63. Model 2, which added baseline assessments of teachers' occupational health and well-being and program expectations, accounted for 21% of the variance and was a significantly better fit for the data than Model 1, *X*^2^(4) = 18.50, *p* < .001, with a negative effect of stress (*B* = −0.40, *SE* = 0.19, *p* = .04) and a positive effect of principal trust (*B* = 0.52, *SE* = 0.16, *p* < .001) driving this improvement. Model 3, which added the interaction of condition by baseline stress, accounted for 27% of the variance and was a significantly better fit than Model 2, *X*^2^(3) = 12.03, *p* < .001, demonstrating that the addition of the interaction of condition by stress significantly improved the model fit over the demographics and main effects models. Thus, Model 3 was the best fit for the data and the effects in this model were interpreted.

**Fig. 2:**
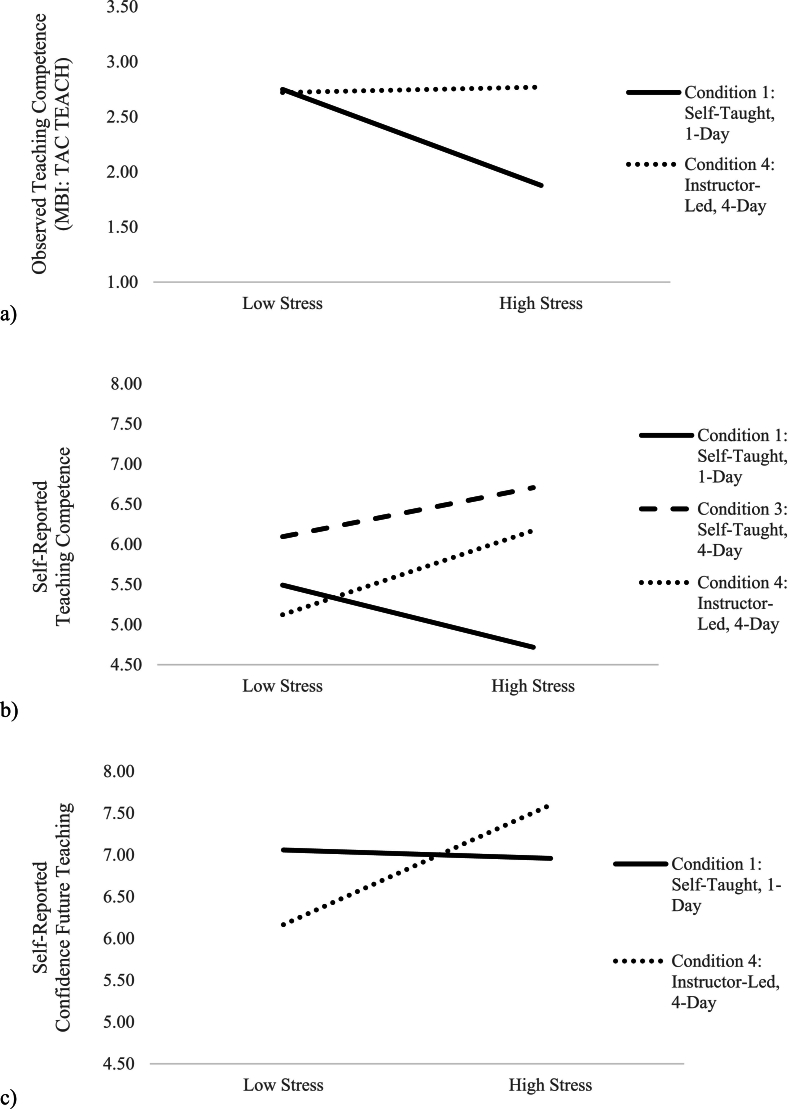
The Effect of Training Condition on Implementation Quality Varied by Teachers' Stress at Baseline. *Note*. Model implied values are displayed. Low/high stress is measured by +/− 1 *SD*. Relatively higher stressed teachers experienced greater quality of implementation across dimensions in Condition 4 (Instructor-Led, 4-Day; displayed in the small dotted lines) than Condition 1 (Self-Taught, 1-Day; displayed in the solid lines).

In Model 3 (Table 6), teachers' trust in their principal at baseline was positively related to observations of quality of implementation (*B* = 0.52, *SE* = 0.16, *p* < .001). The interaction of Condition 4*Stress (*B* = 0.77, *SE* = 0.30, *p* = .01) was also significant. This effect, visualized in Fig. 2a, demonstrates that teachers who were highly stressed (relative to rest of the sample) and who received the most extensive training condition (Condition 4: Instructor-Led, 4-Day) implemented the program with higher quality than did their relatively highly stressed counterparts in Condition 1 (Self-Taught, 1-Day). However, note that the quality of implementation of highly stressed teachers in Condition 1 corresponds to a rating of “beginner” to “advanced beginner” status, still below the desired competency rating of “advanced beginner” and above.

### 3.2 Self-reported teaching competence

Model 1 accounted for 11% of the variance in teachers' self-reported teaching competence, indicating that teachers' gender and training condition explained a modest amount of the variance in this outcome, *X*^2^(4) = 8.45, *p* = .08. Model 2 accounted for 15% of the variance but was not a significantly better fit for the data than Model 1, *X*^2^(4) = 6.56, *p* = .16. Model 3 accounted for 21% of the variance and was a significantly better fit than Model 1 (*X*^2^(7) = 24.54, *p* < .001) and Model 2 (*X*^2^(3) = 11.96, *p* = .01), demonstrating that the addition of the interaction of condition by stress significantly improved the model fit over the demographics and main effects models. Thus, Model 3 was the best fit for the data and the effects in this model were interpreted.

In Model 3, the interaction of Condition 3*Stress (*B* = 1.15, *SE* = 0.50, *p* = .02) and Condition 4*Stress (*B* = 1.52, *SE* = 0.49, *p* = .002) were significant. These effects, visualized in Fig. 2b, demonstrate that the effect of training condition varied by teachers' baseline stress; teachers who were relatively more stressed and who received more training—those in Condition 3 (Self-Taught, 4-Day) and Condition 4 (Instructor-Led, 4-Day)—reported greater competence teaching the course than teachers who were relatively more stressed and received less training in Condition 1 (Self-Taught, 1-Day).

### 3.3 Self-reported confidence in future teaching

Model 1 accounted for a minimal 4% of the variance in teachers' self-reported confidence teaching the course in the future, *X*^2^(4) = 4.77, *p* = .32. Model 2 accounted for 15% of the variance and was a significantly better fit for the data than Model 1 (*X*^2^(4) = 10.03, *p* = .04) with the positive effect of program expectations on confidence teaching driving this improvement, *B* = 0.28, *SE* = 0.10, *p* = .01. Model 3 accounted for 23% of the variance and was a significantly better fit than Model 2 (*X*^2^(3) = 7.99, *p* = .05); thus, the effects from Model 3 were interpreted.

In Model 3, program expectations were positively related to teachers' confidence in future teaching, *B* = 0.27, *SE* = 0.11, *p* = .01. The interaction of Condition 4*Stress (*B* = 1.28, *SE* = 0.52, *p* = .01) was also significant. This effect, visualized in Fig. 2c, demonstrates that although teachers' baseline stress was not related to confidence in future teaching for teachers in Condition 1 (Self-Taught, 1-Day), highly stressed teachers appear to have benefitted the most from the more intensive training of Condition 4 (Instructor-Led, 4-Day), reporting the highest levels of confidence in teaching this course in the future.

## 4 Discussion

Mindfulness-based programs have recently demonstrated promise for supporting students' skills and well-being (Roeser, Galla, & Baelen, 2022; Zenner et al., 2014). Despite evidence from the field of SEL that effective implementation of such programs is critical to achieving theorized outcomes, little research has investigated factors that impact the quality of teachers' implementation of SBMT for students. The present study adds to this new line of inquiry addressing implementation (Sanetti et al., 2020, Sanetti, Dobey and Gallucci, 2014) by investigating multiple factors that impact implementation of a SBMT, specifically whether teachers' feelings of stress, principal and teacher trust, and program expectations at baseline were associated with the quality of teachers' delivery of a SBMT for students. The present study also assessed whether teachers' baseline stress moderated the effect of training condition on implementation quality.

The curriculum was new to all teachers; thus, it was natural for teachers to feel hesitant about teaching a curriculum for the first time, especially as lessons were periodically under observation. Our findings show that teachers who felt more supported by their principals were observed to implement the SBMT with greater quality. In addition, teachers who had more positive expectations about the program felt more confident teaching the course in the future but did not show differences in observed quality of implementation of the program. There was no effect of teacher trust on implementation quality. Importantly, teachers' baseline stress moderated the effect of training condition on all three measures of implementation quality, such that teachers experiencing higher stress at baseline relative to their colleagues implemented the program with greater quality when they received more intensive training in comparison to their highly stressed colleagues who received minimal training. Notably, the present study expands our understanding of implementation in several distinct directions by (a) investigating factors influencing the implementation of a mindfulness-based program for students, whereas most implementation research has focused on other types of SEL programs (e.g., Durlak & DuPre, 2008); (b) focusing on implementation quality, a dimension of implementation not frequently studied in SBMT (Gould et al., 2016); and (c) exploring how characteristics of the program may interact with characteristics of the implementer to impact implementation quality.

### 4.1 Principal support and positive program expectations facilitate implementation quality

The association between principal support and implementation quality indicates that teachers who perceived greater levels of institutional support exhibited greater quality implementation of the SBMT for students. As hypothesized, the present study found that teachers who experienced stronger relationships with their principals implemented the program with higher quality. This study adds to the body of research from SEL and SBMT demonstrating that supportive school leadership facilitates high quality implementation (Han & Weiss, 2005; Joyce et al., 2010; Payne et al., 2006; Ransford et al., 2009; Wilde et al., 2019). Such effects may occur either directly, by principals showing interest in and encouragement of teachers' participation in the program, as described by qualitative research from teachers implementing SBMT (Wilde et al., 2019), or indirectly by creating a climate where teachers feel safe and supported when they take risks implementing novel programs in their classrooms. Interestingly, this association was found with regards to observations of teachers' implementation quality and not their self-reported implementation of the program, indicating that findings cannot be attributed to shared method variance (i.e., self-reported principal support was associated with independent observations of implementation quality). The effect of principal support on implementation quality appears to operate subtly, emerging only in observations of teachers' behaviors in the classroom and not in teachers' subjective self-reports of implementation quality. This suggests that relying on teachers' own subjective ratings of their competence does not convey the whole story.

Findings regarding the effect of program expectations on implementation quality are consistent with other studies demonstrating that teachers who are positively engaged and/or have a positive view of the program at the outset are likely to show more effective implementation of both SEL and SBMT programs (Cook et al., 2015; Han & Weiss, 2005; Ransford et al., 2009; Wilde et al., 2019). Previous research has concluded that program expectations are critical because they are an early indicator of the effort that teachers are willing to invest in the program (Han & Weiss, 2005). These results have important implications for implementation researchers, highlighting that efforts to design SBMT in ways that pique teachers' interest are worthwhile investments.

This study is aligned with the larger movement in implementation science to determine barriers and facilitators to implementation. Although this study focused on the role of teachers' perceived stress, principal and teacher trust, and attitudes about the program in their implementation of a SBMT, a parallel line of work examining clinicians and counselors' implementation of an SEL program has found that personality traits, including agreeableness and contentiousness also impact implementation (Lochman et al., 2015, Lochman, Powell, Boxmeyer, Wells and Windle, 2009). Together, this body of research provides support for Domitrovich et al.'s (2008) conceptual model of factors impacting implementation and highlights the importance of continuing to consider how implementers' background may influence their implementation of programs. This study was grounded in Domitrovich et al.'s (2008) conceptual model because of its tailoring to school-based research and is consistent with broader frameworks of implementation science that have emphasized the importance of the characteristics of implementers (Damschroder et al., 2009).

### 4.2 Highly stressed teachers benefit from more intensive training

The Prosocial Classroom model (Jennings & Greenberg, 2009) posits that stress jeopardizes teachers' ability to effectively implement intervention programs for students. Although results from the present study indicated that highly stressed teachers who received minimal training (in the Self-Taught/1-Day condition) evidenced lower quality implementation, they also demonstrated that highly stressed teachers can implement the program at levels corresponding to their colleagues experiencing relatively lower levels of stress when provided with sufficient training and support (i.e., in the Instructor-Led, 4-Day condition). These results indicate that there were differences in implementation quality between groups, but they do not indicate the specific driver of these effects. It is possible that more intense training in the personal mindfulness and curriculum training resulted in differences in implementation quality; it is also possible that the affective support of a friendly coach afforded in the more intensive training was particularly beneficial for highly stressed teachers. Note however, that the average observed rating for quality of implementation for those highly stressed teachers receiving the more intensive training corresponds to an assessment of “beginner” to “advanced beginner”, with more skilled delivery necessary to reach the level of competent, proficient, and advanced. Although the additional training was helpful, they have not yet met the absolute criteria corresponding to high quality implementation. In sum, these findings emphasize that the program implementer is not blank slate but rather comes to the classroom with background and experiences that influence how they embody and teach the curriculum (Cook et al., 2015; Durlak & DuPre, 2008). In turn, these results highlight the importance of tailoring program training to meet teachers' needs.

Notably, these effects were found with regards to teachers' general levels of global stress. More specific measures of teachers' occupational health, such as their occupational stress, may have even stronger effects than those found here as these experiences are tied to the context in which they were implementing the program. Furthermore, with research demonstrating that teachers' personal well-being (i.e., not tied to the work context), such as depressive symptoms, are associated with poorer quality learning environments (McLean & Connor, 2015), it would also be useful for future research to consider various dimensions of both teachers' occupational health (e.g., occupational stress, burnout) and personal well-being (e.g., depressive and anxiety symptoms) in relation to their implementation of intervention programs.

### 4.3 Limitations and future directions

Although there has been a recent increase in the explicit focus on implementation science, there is still a lot to be learned, particularly with respect to factors impacting implementation of SBMT programs (Baelen et al., 2023; Sanetti et al., 2020, Sanetti, Dobey and Gallucci, 2014). Considering the many advantages of teachers as the implementer of such interventions, the present study focused on the role of teachers' background characteristics with regards to implementation (Chodkiewicz & Boyle, 2017). However, teachers may face additional barriers and facilitators such as time constraints, which were not assessed in this study, but which previous research on SBMT programs has shown to be a concern for teachers (Braun et al., 2020; Joyce et al., 2010). Furthermore, future work should expand our understanding of factors impacting implementation of SBMT by investigating not only the effects of characteristics of the teacher, but also of the school and macro-level factors (Domitrovich et al., 2008). As this study involved teachers in the UK, there may be additional or different factors that are operative in different cultural settings.

It is important to note that the analytic sample in this study included only teachers who participated in post-program data collection. In comparison to teachers who participated in data collection at post-program, those who did not differed in several distinct ways. Suggestions of systematic attrition indicated that missing data techniques were not appropriate for use in these analyses. Thus, the results found in the present study are unique to these teachers and may not generalize to teachers in an intent-to-treat design. In fact, as teachers without post-intervention data had lower principal trust and lower expectations of the program than those with post-intervention data, it is possible that the results in these analyses could have been even stronger should these teachers have had complete data as their inclusion would have resulted in greater variability in the predictors of interest that demonstrated significant associations with implementation quality. We posit that teachers' perceptions of principal trust, teacher trust, and greater program expectations support their sustained engagement in the program that may underlie why teachers with complete data reported higher levels of these constructs. A useful next step would be to investigate reasons for commitment or attrition from the program, which is relevant to the sustainability SBMT over time. Relatedly, the power analyses for the present study indicated that the sample size was just above the threshold needed to detect a medium effect, should it be present. Given these data were already collected, Bacchetti (2010) argued that estimates and significance tests should be emphasized over power analyses as these allow for more direct interpretation of findings. Regardless, future studies with larger sample sizes would be better powered to address more complex research questions and analyses than those used in the present study.

One of the strengths of this study is its use of varied forms of data collection. We were able to dive deeply into implementation quality—a dimension of implementation not often studied in SBMT (Gould et al., 2016)—by analyzing observations and self-reports of teachers' quality of implementation. The use of a single informant has been a noted limitation of previous SBMT studies, of which only 20% included an observational measure of implementation and only 10% used more than one source of data to assess a single dimension of implementation (Gould et al., 2016). Although highly stressed teachers benefitted more from more intensive training across all outcomes, several outcome-specific effects were found for principal support and program expectations. Some research from the field of SEL has demonstrated that observational measures of implementation are more strongly associated with outcomes than self-report measures (Durlak & DuPre, 2008; Lillehoj & Griffin, 2004), yet research on the effect of implementation on outcomes in the context of SBMT is still nascent (Gould et al., 2016). Furthermore, to minimize participant burden, the self-reported measures of implementation quality assessed in this study were single-item measures that have not yet undergone validity and reliability testing. The modest positive correlations between the single-item measures and observed teaching competence suggest that these measures are associated. Additionally, the items have strong face validity, an important yet underutilized assessment of the validity of single-item measures (Allen et al., 2022). Notably, single-item measures are not inherently inferior to multiple-item measures (Allen et al., 2022). Single-item measures assessing teachers' occupational health (e.g., job stress and coping) are known to have meaningful concurrent and predictive associations and have demonstrated sensitivity to intervention (Eddy et al., 2019). Nonetheless, future research should test whether this is the case with the single-item measures used in the present study.

Together with Schultes (2021) and Roeser, Galla, and Baelen (2022), we call for the more intentional study of implementation of SBMT, including the assessment of (a) the different dimensions and reporters of implementation, (b) how these dimensions of implementation are related to program outcomes, and (c) how teachers' personal characteristics and larger macro-level factors may interact with aspects of the program, such as training, to determine how we can best support those playing the critical role of program implementer.

For those beginning to integrate implementation science into research on SBMT, several resources provide conceptual frameworks from which to approach implementation research (e.g., Consolidated Framework for Implementation Research [CFIR], Quality Implementation Framework [QIF]; Damschroder et al., 2009; Domitrovich et al., 2008; Meyers et al., 2012), with the CORE Process specifically tailored to the implementation of SBMT (Gould et al., 2014). Advice regarding selecting measures of implementation and what to do with them (e.g., Schultes, 2021) and practical guidance about how to support implementation in the field are also available (e.g., Flaspohler et al., 2012). Together with this research, the present study suggests that efforts to investigate the barriers and facilitators to implementation of SBMT can lead to greater insight regarding factors that impact the uptake and success of such intervention approaches.

### 4.4 Implications for practice

This study provides several specific insights for practice. First, it highlights that additional support may be necessary when teachers experiencing relatively high levels of stress are implementing SBMTs. This information is likely to be particularly helpful for interventionists and practitioners working in settings known to be associated with high levels of teacher stress, such as schools in under-resourced communities (e.g., Bottiani et al., 2019). Alternatively, some researchers have suggested that to maximize program outcomes, barriers to implementation should be addressed prior to launching the program (Levantini et al., 2021). In other words, an intervention could contain two phases: an intervention to address barriers, then implementation of the intervention. Larson et al. (2018) found this multipronged approach successful such that intervening to reduce teachers' stress improved their subsequent implementation of evidenced-based practices for students. Additional research on the barriers of the specific aspects of implementation most strongly related to outcomes for students will be helpful to ensure such efforts are appropriately targeted.

The multipronged approach is similar to the design of the present project, where Phase 1 was a personal mindfulness training to increase teachers' well-being and Phase 2 was training in the curriculum. Previous results from this project found that the instructor-led personal mindfulness training was associated with greater reductions in stress than the self-taught personal mindfulness training (Montero-Marin, Taylor, et al., 2021), yet the main effect of training condition on implementation quality did not reach significance (Crane, Ganguli, et al., 2020). The present study adds to our understanding of these results by indicating that teachers' implementation varies according to both their stress levels when they begin the program and the type of training they receive, suggesting that more intensive training efforts may be worthwhile in some situations, but also that less intensive training methods may be employed and result in similar levels of implementation quality in others. Understanding the interplay between these factors is relevant to practitioners given that a cost-effectiveness analysis of this program has demonstrated that more intensive training inevitably comes with a greater financial burden (Crane, Ganguli, et al., 2020). Thus, identifying circumstances in which more and less intensive training and coaching is necessary to achieve quality implementation is of practical and economic relevance. At the same time, researchers should be sensitive that additional training for teachers who are already highly stressed may contribute to stigma and increased burden on teachers who are already struggling. Importantly, although intervening at the teacher-level to reduce stress may be helpful in providing teachers with coping strategies, it does not address the larger systemic stressors at the school and district levels that create such a stressful work environment such as a lack of administrator support and rigid accountability assessments (e.g., OFSTED), among others (Case et al., 2000; Haydon et al., 2018; Jerrim & Sims, 2022; Richards, 2012; Saeki et al., 2018).

## 5 Conclusion

The present study investigated whether teachers' experiences of stress, feelings of teacher and principal trust, and expectations about the program at baseline were associated with the quality of their implementation of a SBMT for students and whether teachers' baseline stress moderated the association between training condition and implementation quality. Results demonstrated that teachers' feelings of principal support at baseline and positive engagement with the program at an early stage were associated higher quality implementation. Furthermore, although highly stressed teachers have been posited to be at risk for poor quality implementation of intervention programs for students, this study found that relatively highly stressed teachers demonstrated commensurate levels of quality of implementation when they were provided with more intensive training in both personal mindfulness and the curriculum for students. These results emphasize that program implementers should be recognizing as active participants in the intervention process and highlight that implementers may vary in the extent of training needed to achieve high quality implementation.
